# Urinary incontinence in men with Duchenne and Becker muscular dystrophy

**DOI:** 10.1371/journal.pone.0233527

**Published:** 2020-05-29

**Authors:** Christopher I. Morse, Katie Higham, Emma L. Bostock, Matthew F. Jacques

**Affiliations:** 1 Department of Sport and Exercise Sciences, Research Centre for Musculoskeletal Science & Sports Medicine, Manchester Metropolitan University, Manchester, United Kingdom; 2 Musculoskeletal Physiology Research Group, School of Science and Technology, Nottingham Trent University, Nottingham, United Kingdom; 3 School of Life Sciences, Queens Medical Centre, University of Nottingham, Nottingham, United Kingdom; University Medical Center Utrecht, NETHERLANDS

## Abstract

The prevalence of urinary incontinence in Duchenne and Becker muscular dystrophy (D/BMD) is reported to be between 15–29%, this however includes ages across the lifespan, and with no description of impact on daily life. The present study, aimed to determine the prevalence of urinary incontinence in men with D/BMD, and to identify which aspects of daily life were impacted by urinary incontinence. Twenty-seven adult males, 11 with BMD and 16 with DMD, aged 20–57 years, volunteered to participate in this study. Six questionnaires were completed to provide an overview of participant mobility, urinary incontinence and distress caused by urinary incontinence. These included: The Barthel index of disability, International Consultation on Incontinence Questionnaire—Urinary Incontinence Form, Incontinence Impact Questionnaire Short Form, The Urogenital Distress Inventory, and the Brooke and Vignos scale. The prevalence of urinary incontinence within the present men with D/BMD was 37%, assessed as urine leakage of once a week or more. Those with urinary incontinence all reported only a “small amount” of urine leakage, with urinary incontinence frequency of once a week in 5/10 participants, two or three times a week in 2/10 participants, and once a day in 3/10 participants. Of those with urinary incontinence 8/10 experienced some impact on their daily life from urine leakage including travel (4/10), social aspects (5/10), and emotional aspects (8/10). All participants with urinary incontinence were bothered by some aspect, including urine leakage (9/10), and frequent urination (4/10). In conclusion, 37% of the present men with D/BMD experience urinary incontinence on a weekly or daily basis and negatively impacted aspects of life related to travel, social and emotional wellbeing. Urine leakage, and frequent urination should be considered a meaningful problem by care providers, and discussed openly with those with D/BMD.

## Introduction

Duchenne and Becker muscular dystrophy (D/BMD) are progressive muscle wasting conditions characterised by the absence or reduced expression of the cytoskeletal protein dystrophin [1]. DMD, characterised by absent or non-functional dystrophin, is the most severe form of dystrophinopathy, with diagnosis in early childhood, and a progressive decline in muscle function and mobility [2]. BMD presents with partially functioning dystrophin, and is therefore a milder yet more variable form of dystrophinopathy [2], with about 50% of adults with BMD maintaining the ability to walk [3]. Despite the well-established decline in skeletal muscle strength being the cause of a loss of mobility and ambulation in D/BMD, there is only limited evidence that skeletal muscle weakness contributes to a lower quality of life (QoL) in adults with D/BMD [4].

The pathology and impact of skeletal muscle weakness in adults with D/BMD has become more relevant as life expectancy has increased, particularly for those with DMD who can now live well into adulthood [5]. It is however apparent that despite skeletal muscle weakness being a primary clinical sign, the impact of D/BMD on smooth muscle may contribute to the presentation of co-morbidities, particularly within the urinary tract [6]. In boys with DMD, there is a 50% prevalence of lower urinary tract symptoms (LUTS) [7], which could in part be linked to the expression of dystrophin within the smooth muscle of the bladder and upper urinary tract [8]. Ambiguity surrounding the reporting of LUTS (i.e. inconsistent symptoms being reported) and the inclusion of mixed age groups with D/BMD, may contribute to broad prevalence of LUTS being reported at between 50% and 90% [9, 10].

It is however, urinary incontinence (UI) rather than LUTS that seems to impact daily life. Indeed, in males aged 4–33 yrs with D/BMD, LUTS are reported by 71% but only 29% consider them to be “bothersome” [9]. By coincidence in the aforementioned study, UI was reported in 29% of participants [9], a value which may be skewed by the inclusion of a high proportion of younger children, who the authors acknowledge may not be toilet trained, and whom the impact of impaired skeletal muscle may not necessarily result in UI. In men with DMD (>19 yr olds) urinary incontinence is reported at 15% when classified using a yes or no response, despite almost 90% reporting one or more LUTS [10].

The subjective measurement of incontinence severity through reporting life impact and symptom distress are important in understanding the patient related outcomes associated with urinary incontinence [11]. In D/BMD, the condition itself already impacts on daily life with men with DMD and BMD experiencing lower QoL in the mental health, social and emotional domains compared to adults without MD [4]. The impact on daily life of UI seems to be quite low based on the mixed age group DMD data (Males aged 4–40 yrs), with only 9% experiencing limitations in social life [10]. It would be expected however that the impact of UI would be very different in adult populations compared to children and adolescents, given the progressive muscle weakness associated with D/BMD. The Incontinence Impact Questionnaire Short Form (IIQ-7) distinguishes the impact of incontinence on the domains of daily life such as social, emotional and travel [11]. The urogenital distress inventory (UDI) was developed to complement the IIQ-7 and to identify whether participants are “bothered” by symptoms of UI [11]. To our knowledge, neither IIQ nor UDI have previously been reported in men with D/BMD.

Given the broad range previously reported for the prevalence of UI (15–29%), the range and mixture of ages, and the lack of reported impact on daily life in men with D/BMD, the present study, aimed to determine the prevalence of UI in men with D/BMD, and to identify which aspects of daily life were impacted by UI.

## Materials and methods

Twenty-seven adult males, 11 with BMD and 16 with DMD volunteered to participate in this clinical epidemiological study (participant characteristics are provided in Table 1). All participants were recruited face-to-face at the Neuromuscular Centre (NMC, Winsford, UK), or via posting through the NMC social media pages. All participants completed an online questionnaire (JISC, UK), those recruited face-to-face were sent the link directly, whilst those recruited via social media could click through from the post itself. The landing page of the questionnaire provided participant information, with a consent to participate drop down selection. Those providing consent to participate were directed to the first page of the questionnaire, those who chose not to consent were directed to an exit page for the survey. All procedures were approved by the ethics committee of Manchester Metropolitan University, UK.

**Table 1:** Demographic and questionnaire outcomes from men with Duchenne Muscular Dystrophy (DMD) and Becker Muscular Dystrophy (BMD) who experienced at least weekly urinary incontinence (incontinent) or no incontinence (continent).

	All	Continent	Incontinent
n	27	17	10
DMD/BMD	11/16	10/7	4/6
Age (years)	31.4 (9.6)	30.5 (10.5)	32.9 (8.2)
Brooke	6 (1–6)	5 (1–6)	6 (1–6)
Vignos	9 (2–9)	9 (2–9)	9 (2–9)
BI (-UI)	5.9 (6.0)	6.2 (6.0)	5.3 (6.3)
ICIQ Total	2.1 (3.2)	0.4 (1.5)	5.8 (2.6)†
ICIQ Interference	0.7 (1.5)	0 (0)	2.0 (1.9) †
IIQ-7_SF	6.2 (10.8)	0.8 (3.4)	15.3 (13.1) †
IIQ Physical	1.9 (7.1)	0 (0)	5.0 (11.2)
IIQ Travel	5.6 (13.1)	2.0 (8.1)	11.7 (17.7)*
IIQ Social	9.9 (20.3)	2.0 (8.1)	23.3 (27.4)*
IIQ Emotional	9.3 (17.5)	0 (0)	25.0 (21.0)†
UDI-6	12.1 (12.8)	5.2 (7.4)	20.8 (13.0) †
NBD	3.52 (3.95)	3.18 (3.54)	4.10 (4.70)

Questionnaires include: The Barthel index (BI) of disability (-UI, minus urinary incontinence score), International Consultation on Incontinence Questionnaire—Urinary Incontinence Form (ICIQ-UI_SF), Incontinence Impact Questionnaire Short Form (IIQ-7_SF), The Urogenital Distress Inventory (UDI-6), and The Neurogenic bowel dysfunction (NBD) score. Data are median (minimum-maximum), or mean (SD).

* and ^†^ denotes significant difference from Continent participants, P<0.05 and P<0.01, respectively.

In total six questionnaires were included to provide an overview of participant mobility, UI and distress caused by UI. These included: The Barthel index (BI) of disability, International Consultation on Incontinence Questionnaire—Urinary Incontinence Form (ICIQ-UI_SF), Incontinence Impact Questionnaire Short Form (IIQ-7_SF), The Urogenital Distress Inventory (UDI-6), and the Brooke and Vignos scale. A seventh questionnaire, the Neurogenic Bowel dysfunction score was included for descriptive purposes of bowel dysfunction.

### The Brooke and Vignos scale

The Brooke [12] and Vignos [13] scales are used to assess the upper and lower limb functions, respectively. These scales have been previously used when measuring functional status in DMD/neuromuscular diseases [14]. The Brooke scale ranges from 1–6, with 1 meaning the participant is able to “start with arms at the sides and can abduct the arms in a full circle until the touch above the head”, and 6 “Cannot raise hands to the mouth and has no useful function of hands”. The Vignos scale ranges from 1–10, with 1 being able to “Walk and climb stairs without assistance”, and 10 “Confined to a bed”. Higher scores indicate a greater level of impairment.

### The Barthel Index (BI)

The BI is a disability specific, 10-item questionnaire to self-assess performance of activities of daily living [15]. The BI scoring system ranges from 0–20, with lower scores indicating increased disability. The full questionnaire is scored out of 100, and is presented as such in the population description. As incontinence is included as a single question within the BI, these data are omitted where comparisons are made between the continent and the incontinent participants, in this instance the score is presented out of 80.

### ICIQ-UI SF

The four item ICIQ-UI SF questionnaire was used to determine current UI status, volume of urine that leaks, and a single question on how urine loss may interfere in everyday activities [16]. Scoring is 0–21 with higher scores indicating greater severity of UI, and daily impact. Data for the population as a whole is presented on a scale of 0–21, however each of the three questions are considered separately for comparison between incontinent and continent participant groups. Specifically, Q1-how often does urine leak-was used to classify UI. Q2-was used to determine how much urine leaks; and Q3-provided a level of interference with everyday life, where 0 = not at all and 10 = a great deal.

### IIQ-7 SF

The IIQ-7 SF, is a seven item questionnaire assessing the impact of UI on aspects of quality of life [11]. The questions pertain to physical activity, travel, social/relationships, and emotional health. Item responses were scored 0 for "not at all," 1 for "slightly," 2 for "moderately," and 3 for "greatly." The average score of items was calculated and multiplied by 33.3 to put scores on a scale of 0 to 100.

### UDI-6

The UDI-6 is used to assess symptoms of distress and whether participants are “bothered” by aspects of UI [11]. The scoring system of the six questions ranges from 0–3, a higher number indicates UI to be bothersome. Item responses were scored 0 for "not at all," 1 for "slightly," 2 for "moderately," and 3 for "greatly." The average score of items was calculated and multiplied by 33.3 to put scores on a scale of 0 to 100.

### Neurogenic Bowel Dysfunction (NBD)

The focus of the present study is UI, for completeness however, bowel dysfunction was recorded using the NBD questionnaire. The NBD consists of 10 questions on aspects of flatus, faecal incontinence, constipation and defecation, with a total score of 0–47, where a higher score indicates a more severe bowel dysfunction [17]. Participants were classified by severity of NBD as follows: 0–6 = Very minor, 7–9 = minor, 10–13 = moderate, >14 = severe.

### Classification of UI

Participants were classified as incontinent based on their replies to the following question within the ICIQ-SF: “In the last 4 weeks, how often have you leaked urine?” Those responding as leaking “about once a week or less often”, “two or three times a week”, “about once a day”, “several times a day” or all the time”, were classified as incontinent. This definition is similar to others within the general population who have classified UI as “any urine leakage twice or more a month” [18, 19], or much broader definitions as “any involuntary loss of urine” [20]. Where relevant, data is first presented as a score including all question responses, but when segregated into incontinent and continent, some questions are omitted if a score is based on urine leakage (e.g. BI Q6).

### Statistics

All quantitative analyses were performed using IBM SPSS Statistics 24 software. Based on all grouped data showing non-normal distribution using Shapiro-Wilk’s test (P<0.05), and similar conclusions being drawn when Likert scales are assessed as parametric or non-parametric [21], all data were considered non-parametric. The Mann-Whitney U test was used for group comparisons between continent and incontinent groups. It should be noted however, that despite continent and incontinent comparisons being reported in our data, they are not discussed in detail, as greater meaning is derived from interpreting the level of impact and interference in those with UI. Spearman’s rank correlation was performed to determine whether correlations between lower limb impairment were associated with severity of UI symptoms e.g. UDI totals.

Data are presented as median (range) for single Likert scales (e.g. Brooke and Vignos), and Mean (SD) where totals (BI, ICIQ-UI, NBD), or averages (IIQ-7, UDI-6) have been used to calculate overall scores within each questionnaire.

## Results

The prevalence of UI within the present men with D/BMD was 37%, with urine leakage of once a week or more reported by 10 of the 27 participants. Compared to the continent participants, those with UI showed no difference in age, Brooke score, Vignos score, or BI activities of daily living (Table 1).

Based on the data from the ICIQ (Table 1), of those classified as UI all reported only a “small amount” of urine leakage, with UI frequency reported as once a week in 5/10 participants, two or three times a week in 2/10 participants, and once a day in 3/10 participants.

In terms of interference with everyday life, the ICIQ and the IIQ revealed that overall, 8/10 of those with UI experienced some impact on their daily life (Table 2). The IIQ showed that urine leakage interfered with travel in 4/10 UI participants, social aspects in 5/10 UI participants, emotional aspects in 8/10 UI participants and physical aspects in 2/10 UI participants. The severity of urine leakage impact in UI participants was highest for emotional and social aspects (Table 1), with lesser impact on travel and physical aspects (the latter showing no difference between UI and continent participants, Table 1). Based on the ICIQ, urine leakage was found to occur before they can reach a toilet in 5/10 participants, post urination when dressed in 5/10 participants, when asleep in 3/10 participants, when coughing or sneezing in 2/10, and when exercising in 1/10 participants.

**Table 2:** Incontinence Impact Questionnaire Short Form (IIQ-7_SF). Incontinence impact from all men with Duchenne and Becker muscular dystrophy.

	Not at all	Slightly	Moderately	Greatly
Chores	26 (96%)	0	1 (4%)	0
Physical Activity	26 (96%)	1 (4%)	0	0
Entertainment	22 (82%)	3 (11%)	2 (7.4%)	0
Travel	25 (93%)	2 (7%)	0	0
Social	21 (78%)	4 (15%)	2 (7%)	0
Emotional	22 (82%)	3 (11%)	2 (7%)	0
Frustration	20 (74%)	6 (22%)	1 (4%)	0

Data are presented as: Participant numbers (Percentage of total)

The UDI revealed that all participants with UI were bothered by some aspect of UI, with the overall UDI score higher than those without UI (P<0.01, Table 1). Urine leakage was the most bothersome (9/10 participants), followed by frequent urination (4/10 participants, Table 3). Based on the outcomes of the UDI, 5/10 participants would be classified as having urge or frequency incontinence, 2/10 as having stress incontinence, and 1/10 as having mixed incontinence. There was no association between UDI severity and either Vignos scale (r = 0.22) or Barthel index (r = -0.143). Toilet use independent of help was reported by 30% of participants with UI, and 38% of those without UI. The Barthel index score for toilet independence was not different between those with and without UI.

**Table 3:** The Urogenital Distress Inventory (UDI-6). The amount of bother due to symptoms of urinary incontinence in all men with Duchenne and Becker muscular dystrophy.

	Not at all	A little bit	Moderately	Greatly
Frequent urination	17 (63%)	3 (11%)	4 (15%)	3 (11%)
Leakages from urgency	22 (82%)	3 (11%)	2 (7%)	0
Leakage from cough/sneeze	25 (93%)	2 (7%)	0	0
Urine leakage	17 (63%)	7 (26%)	3 (11%)	0
Difficulty emptying bladder	20 (74%)	4 (15%)	2 (7%)	1 (4%)
Pain in abdomen	22 (82%)	4 (15%)	1 (4%)	0

Data are presented as: Participant numbers (Percentage of total)

There was no difference in NBD between those with and without UI (Table 1). Overall, all but four participants were classified as “very minor” in terms of NMD severity, with 1 classified as “minor”, and the other 3 as “moderate”. There was no group difference in the prevalence of participants taking treatment for constipation (50% of those with UI and 47% of those without UI).

## Discussion

The present study aimed to determine the prevalence of UI in men with D/BMD, and to identify which aspects of daily life were impacted by UI. The main findings were that 37% of men with D/BMD could be considered incontinent, with all of those with UI experiencing some interference or bother to daily life. The most frequent and severe impact to daily life was reported in social and emotional aspects.

The prevalence of UI in the present study compared to men without D/BMD can only be made by considering the definition of UI. Despite numerous definitions used to define UI within the literature (all those that follow are self-reported, consistent with the present study), the prevalence of UI in the present men with D/BMD is higher. UI in adult men without MD is reported as 13% (any leak in 12 months, men aged 30–49 [22]), 5% (urine leakage >twice/month, men aged 42–78 [19], and 0.8% (>twice/month, men aged 25–35 [18]). Of course this is a selection of examples where men are used, age, and definition are provided, however there are a multitude of other data available [23], but throughout all of the similar demographic groups, the prevalence of UI (37%) is considerably higher in the present men with D/BMD.

Previous data on UI prevalence in D/BMD has tended to focus on boys with DMD; with UI prevalence of 25% [7] and 29% [9] reported previously. These previous studies do not however, provide a distinction between ages, despite ranging from 3–25 yrs and 4–33 yrs, respectively. Where UI prevalence has been reported in men with D/BMD [10], the 15% prevalence of UI is considerably lower than the 37% in the present study, or the 25–29% from those studies including children [7, 9]. It is likely that the dichotomous (yes/no) classification of the self-reported data from the previous study [10], may be associated with underreporting by their participants. Our present UI prevalence would for example be much lower, should those participants who reported UI leakage of once a week, not consider themselves as incontinent had we used a dichotomous classification in the present study. Regardless of the difference of classification of UI between the present and previous men with D/BMD [10], here we have specifically identified novel interference of daily life in travel, emotional and social aspects as a result of UI.

Based on the UIIQ, and UDI, men with D/BMD who have UI all experience a negative impact on their daily life. The UIIQ revealed that travel, social and emotional aspects of daily life were negatively impacted by UI in men with D/BMD. With urine leakage and frequent urination being the most bothersome aspects of UI. As we have reported previously, QoL is lower in those with BMD and DMD compared to non-MD controls [4]. Based on our present data of urinary impact and distress, despite not recording QoL directly in the present study, UI undoubtedly has a negative impact on daily life in a considerable proportion (i.e. 37%) of men with D/BMD.

In the present population of men where mobility is severely impaired, the high prevalence of UI could be attributed to issues with mobility, such that they cannot reach the toilet prior to urine leaking. In the present data we saw no associations between lower limb function and UDI severity, no difference in lower limb function between those with and without UI, and no difference in the independence of toilet use in those with and without UI. That being said, 50% of those with UI did report that urine leaks before they can reach the bathroom. It may however not be mobility, but issues of access to specialist facilities, availability of assistance, or indeed, overcoming stigma to request help such as in other populations [24].

In the present study, in the absence of previous data, we report UI prevalence, impact and distress in men with D/BMD. It is however, only through considering coping strategies, potential interventions, and assistance from carers that those with DMD, and BMD, could experience lower impact on their daily life. In the present study, we identify which aspects of daily life are impacted by UI, making it possible to understand where support is needed for men with D/BMD. In other groups where UI is prevalent, numerous studies describe pathways to help and coping strategies [25], managing UI through the description of lived experiences [26], and the potential benefit of pelvic floor muscle training [27]. Yet in men with D/BMD, we have found no description of any aspect of treatment, coping or management of UI, despite its high prevalence. It is through understanding how the lives of those with D/BMD are impacted, that control of treatment and care can be provided to those with the condition, and allow them to engage meaningfully with the process [28]. The data presented by van Wijk, Messelink (10) emphasises a meaningful point, and although not explicitly discussing the comparison themselves, they show that of 199 participants with DMD (4–40 yrs), 170 report LUTS, yet only 44 have mentioned LUTS to their doctor. It is also noticeable that there is no mention of UI or similar issues in the seminal description of DMD management [29], nor is it a topic presently addressed by the leading UK charity and information provider for those living with D/BMD, Muscular Dystrophy UK (aside from a blog post by those with MD, experiencing UI).

In contrast to previous description of men with DMD (n = 52, [10]), our combined D/BMD participant group represents a smaller description of those with the condition (n = 27). The issues of using a smaller sample are somewhat mitigated by focusing our study on the descriptive, rather than inferential comparisons between two groups (although data is presented where relevant). We therefore present our results with the caveat that the overall prevalence of UI within men with D/BMD may be subject to the variance of our modest population. Here we should emphasise that in the presence of no previous data on UIIQ or UDI in men with D/BMD our data is a meaningful contribution to the description of a co-morbidity that is underreported and clearly has a negative impact on daily life that requires further focus from clinicians and researchers.

In conclusion, a substantial proportion of men with D/BMD experience UI on a weekly or daily basis. In a group of men where QoL has previously been reported to be lower than those without MD, UI was found to negatively impact aspects of life related to travel, social and emotional wellbeing. Urine leakage, and frequent urination were also found to be bothersome to those with UI, suggesting a meaningful problem that needs to be considered by care providers, and discussed openly with those with D/BMD.

## Supporting information

S1 Data (XLSX)
